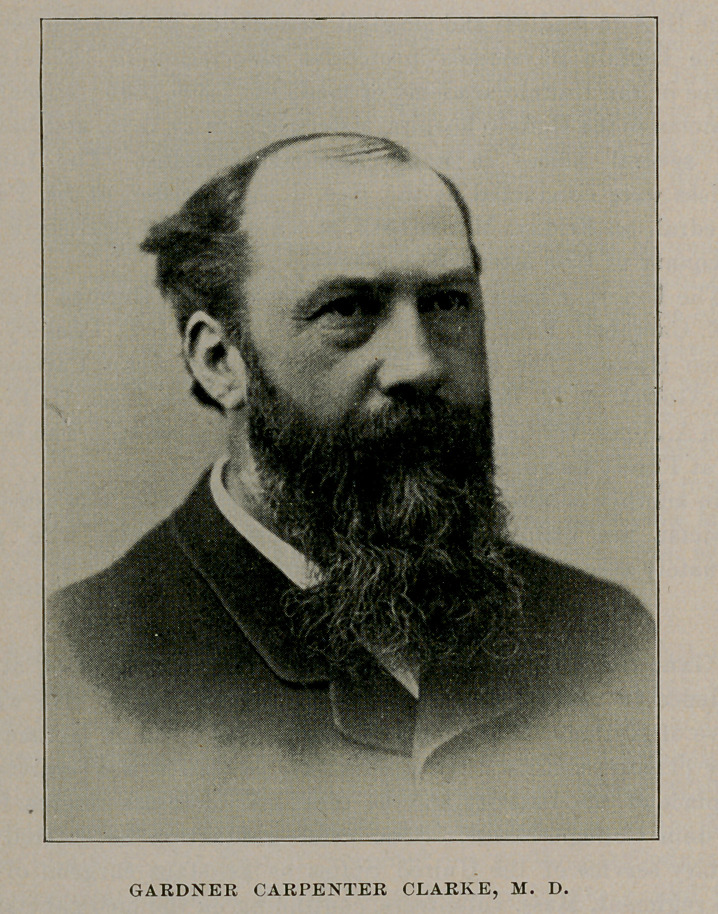# Dr. Gardner Carpenter Clarke

**Published:** 1897-04

**Authors:** 


Dr. Gardner Carpenter Clarke, of Niagara Falls, died at his residence in that city, March 22, 1897, aged 56 years. He was a native of Rutland, Mass., born March 15, 1841, son of Horace and Mary (Tenny) Clarke. His preliminary education was received at the place of his nativity and he took his doctorate degree from Bowdoin College, Maine. Soon after graduation he entered the military service of the United States as assistant surgeon of the 10th regiment, Mass. volunteers, continuing on the medical staff of the army until the close of the civil war in 1865.

Dr. Clarke came to Niagara Falls in 1865, where he has been engaged in the active practice of his profession until a few days before his death. He married Ella M. Granger, of Pittsfield, Mass., in 1866, and she died in November, 1889. He is survived by three children, one a son, Edward G. Clarke, who is manager of the Ontario silver company, at Muncie, Ind., and two daughters,

Elizabeth G. and Mary G. Clarke, who reside at the family home at Niagara Falls.

Dr. Clarke was recognised as the foremost physician of his region, where he had been a leader in professional and social ranks for many years, advancing from obscurity to renown, and from comparative poverty to wealth and influence. He ministered unto the

GARDNER CARPENTER CLARKE, M. D.

poor and rich alike, always rendering his best service whether the prospective compensation was a liberal fee from a competent purse or a simple "God bless you" from an honest heart. He was well known to all the inhabitants of Niagara Falls as a liberal-minded, large-hearted and able physician, who commanded the respect of his professional colleagues as well as the confidence and esteem of the entire community.

Dr. Clarke took as an associate in 1880 Dr. W. R. Campbell, which partnership continued until they were separated by death. Dr. Campbell's kindly care and faithful ministrations were a tender memory in Dr. Clarke's last days.

The funeral was held at the family residence, Wednesday afternoon, March 24th, at 3.30 o'clock, and was largely attended by relatives, friends, neighbors, citizens and his professional colleagues. The flag over the liberty pole floated at half-mast on the day of the funeral out of respect to Dr. Clarke's memory. The interment was at Oakwood cemetery.

## Figures and Tables

**Figure f1:**